# Evidence generation for diabetes prevention and treatment through the integration of clinical medicine, nutritional epidemiology, and behavioral science

**DOI:** 10.1007/s13340-026-00948-9

**Published:** 2026-11-10

**Authors:** Megu Y. Baden

**Affiliations:** 1 Department of Metabolic Medicine, Graduate School of Medicine, The University of Osaka, 2-2 Yamadaoka, Suita, Osaka, 565-0871 Japan; 2 Department of Lifestyle Medicine, Graduate School of Medicine, The University of Osaka, 2-2 Yamadaoka, Suita, Osaka, 565-0871 Japan

**Keywords:** Diabetes prevention, Diet quality, Nutritional epidemiology, Dietary therapy, Ectopic fat, Eating behavior

## Abstract

Diet and other lifestyle factors are central to diabetes prevention and management. Effective dietary and lifestyle support requires consideration of differences in pathophysiology, food culture, and living circumstances. This narrative review summarizes our research integrating clinical medicine, nutritional epidemiology, and behavioral science, with a particular focus on diet quality. Analyses of large US cohorts showed that higher-quality plant-based diets were associated with lower risks of stroke and mortality. In longitudinal biomarker analyses, increases in healthful plant-based diet scores were associated with favorable changes in high-sensitivity C-reactive protein, whereas increases in unhealthful plant-based diet scores were associated with unfavorable changes in inflammatory biomarkers. Metabolite profiles related to healthful plant-based diets were also associated with lower type 2 diabetes risk. Building on earlier work on adiponectin and type 1 diabetes, our studies in Japan showed that even small weight changes were associated with type 2 diabetes risk, depending on age and baseline body mass index. Visceral fat correlated with ectopic fat, and fast eating and eating late at night were associated with greater pancreatic fat deposition. In older adults, lower sweet taste sensitivity was associated with frailty and poorer mental health-related quality of life. These findings suggest that diet quality and lifestyle should be assessed together with each person’s metabolic characteristics and living circumstances. Intervention and implementation studies based on this largely observational evidence are the next step toward individualized diabetes prevention and care.

## Introduction

Improving diet and other lifestyle factors is fundamental to the prevention and management of diabetes. The Diabetes Prevention Program, a randomized trial conducted in the United States, enrolled 3,234 adults without diabetes who had elevated fasting and post-load plasma glucose concentrations. An intensive lifestyle intervention targeting weight reduction and increased physical activity reduced the incidence of type 2 diabetes by 58% compared with placebo over a mean follow-up of 2.8 years [1]. This trial demonstrated that structured lifestyle support can prevent or delay the onset of type 2 diabetes in people at high risk.

Applying such evidence in Japan requires consideration of population characteristics. East Asian populations, including Japanese people, tend to develop type 2 diabetes at a lower body mass index (BMI). Lower insulin secretory capacity and a greater tendency to accumulate visceral fat are thought to contribute to this pattern [2, 3]. Food culture and social circumstances also influence the feasibility of lifestyle changes. Evidence generated in Japanese populations is therefore needed to develop dietary and lifestyle recommendations tailored to their metabolic characteristics and living circumstances.

We have conducted nutritional epidemiologic studies of diet and long-term health outcomes in large US cohorts, as well as clinical studies of diabetes pathophysiology and lifestyle in Japanese populations. This narrative review summarizes these studies, with an emphasis on diet quality, and considers how clinical medicine, nutritional epidemiology, and behavioral science can inform diabetes prevention and care.

## Diet quality, lifestyle, and health outcomes in nutritional epidemiology

Nutritional epidemiology investigates relationships between diet, related behaviors, and health. Its scope has expanded from individual nutrients and foods to overall dietary patterns. At the Department of Nutrition, Harvard T.H. Chan School of Public Health, I examined these associations using large US cohorts, including the Nurses’ Health Study, Nurses’ Health Study II, and Health Professionals Follow-up Study. Repeated dietary assessments in these cohorts allow investigation of both habitual intake and changes in diet over time.

**Fig. 1:**
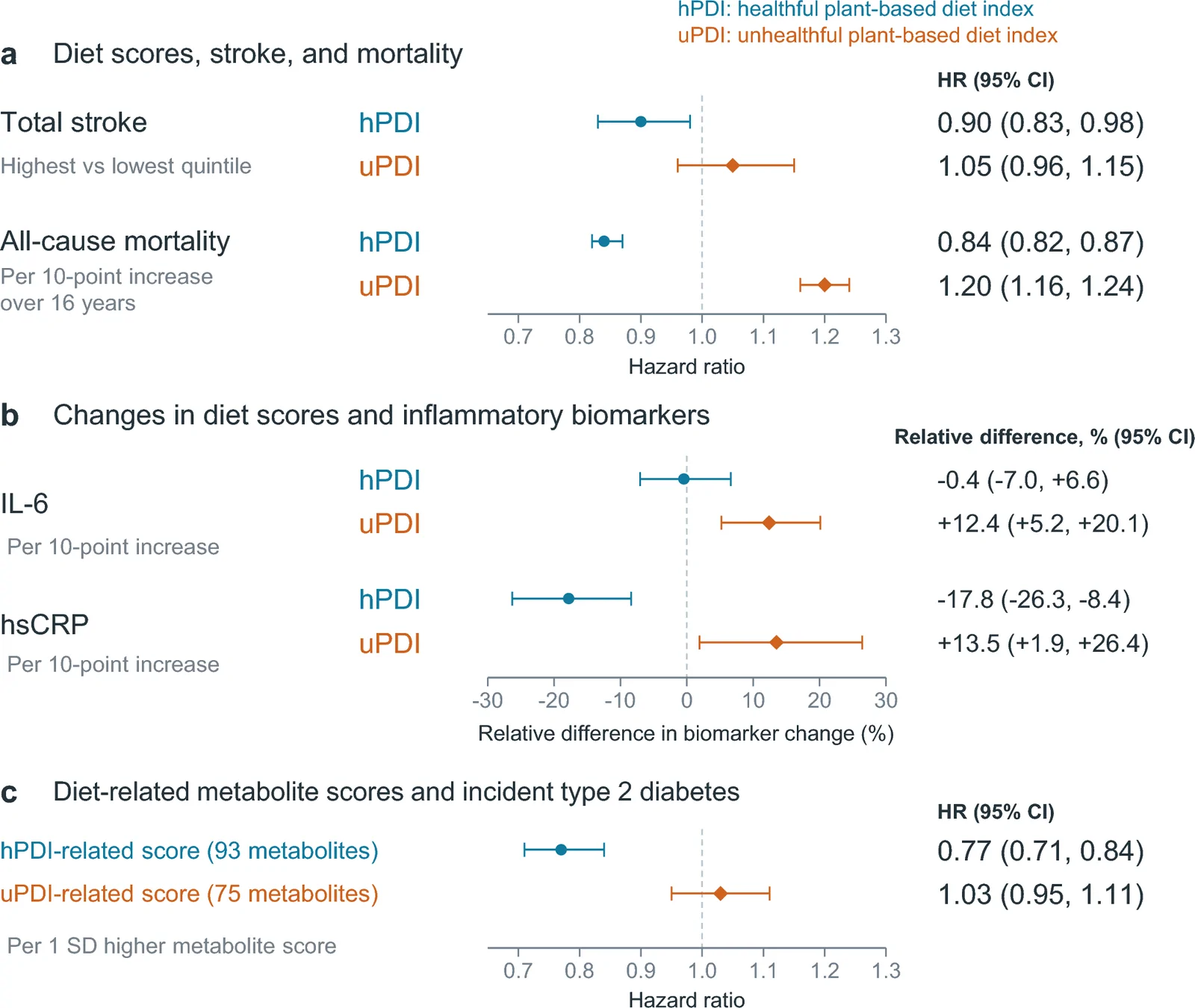
Plant-based diet quality and health outcomes. **a** Hazard ratios (HRs) for total stroke (highest vs. lowest quintile) and all-cause mortality (per 10-point increase over 16 years). **b** Relative differences (%) in changes in interleukin-6 (IL-6) and high-sensitivity C-reactive protein (hsCRP) per 10-point increase in dietary scores. **c** HRs for type 2 diabetes per 1-SD higher metabolite score. Points and horizontal lines indicate estimates and 95% confidence intervals (CIs). hPDI, healthful plant-based diet index; uPDI, unhealthful plant-based diet index; SD, standard deviation. Based on data from references [4–7]

The quality of plant foods is an important distinction when evaluating plant-based diets. Whole grains, vegetables, legumes, and nuts differ from refined grains, sugar-sweetened beverages, and sweets in their nutritional quality and associations with health outcomes. The healthful plant-based diet index (hPDI) and unhealthful plant-based diet index (uPDI) distinguish these food groups. The hPDI gives higher scores to greater consumption of healthful plant foods and lower consumption of less healthful plant foods and animal foods. The uPDI gives higher scores to greater consumption of less healthful plant foods and lower consumption of healthful plant foods and animal foods. Thus, both indices reward lower intake of animal foods but differ in the quality of plant foods they reward. These measures enabled us to investigate how plant-based diet quality relates to clinical outcomes and biological markers (Fig. 1).

## Plant-based diet quality and risks of stroke and mortality

Among 209,508 participants in the Nurses’ Health Study (NHS), Nurses’ Health Study II (NHS II), and Health Professionals Follow-up Study (HPFS), higher hPDI scores were associated with a lower risk of total stroke [4]. The multivariable-adjusted hazard ratio (HR) comparing the highest with the lowest quintile was 0.90 (95% confidence interval [CI] 0.83–0.98). The corresponding estimate for the uPDI was 1.05 (95% CI 0.96–1.15), which was not statistically significant (Fig. 1a).

We also examined changes in plant-based diet quality and subsequent mortality among 75,314 participants in the NHS and HPFS [5]. In an additional analysis of dietary changes over 16 years, each 10-point increase in the hPDI was associated with 16% lower all-cause mortality (HR 0.84, 95% CI 0.82–0.87), whereas each 10-point increase in the uPDI was associated with 20% higher mortality (HR 1.20, 95% CI 1.16–1.24; Fig. 1a). The findings show that changes in the quality of plant foods consumed are related to long-term health.

## Inflammatory biomarkers and metabolite profiles

To explore biological pathways that might underlie these associations, we examined changes in diet quality and plasma biomarkers in 831 women in the NHS II with repeated blood samples approximately 13 years apart [6]. After adjustment for weight change and other covariates, a 10-point increase in the hPDI was associated with a 17.8% lower relative change in high-sensitivity C-reactive protein (hsCRP; 95% CI − 26.3% to − 8.4%). Each 10-point increase in the uPDI was associated with higher relative changes in hsCRP and interleukin-6 (IL-6), by 13.5% (95% CI 1.9–26.4%) and 12.4% (95% CI 5.2–20.1%), respectively. The association between change in the hPDI and change in IL-6 was not statistically significant (Fig. 1b).

In a complementary metabolomics study of 10,684 participants in the NHS, NHS II, and HPFS, we identified plasma metabolite profiles associated with plant-based dietary patterns and constructed hPDI-related and uPDI-related metabolite scores comprising 93 and 75 metabolites, respectively [7]. In prospective analyses, a one-standard-deviation (SD) higher hPDI-related metabolite score was associated with a 23% lower risk of type 2 diabetes (HR 0.77, 95% CI 0.71–0.84). The uPDI-related metabolite score was not significantly associated with diabetes risk (HR 1.03, 95% CI 0.95–1.11; Fig. 1c). These results help characterize the metabolic features of healthful plant-based diets and provide insight into potential mechanisms linking these diets to lower diabetes risk. Metabolite profiles may also complement self-reported dietary assessment.

## Healthy dietary patterns and all-cause mortality

A study of 119,315 participants in the NHS and HPFS followed for up to 36 years compared four dietary scores: the Healthy Eating Index-2015, the Alternate Mediterranean Diet score, the hPDI, and the Alternate Healthy Eating Index [8]. Greater adherence to each pattern was associated with lower all-cause mortality. The consistent associations across multiple dietary scores support dietary recommendations that emphasize overall diet quality while allowing flexibility in food choices to accommodate individual preferences and cultural backgrounds.

## Sleep duration trajectories and type 2 diabetes risk

Sleep is another potentially relevant component of diabetes prevention. Among 60,068 women in the NHS II, we examined sleep duration trajectories from early to middle adulthood in relation to subsequent type 2 diabetes [9]. Compared with a stable sleep duration of 7 h, persistently short sleep and increases or decreases in sleep duration were associated with higher diabetes risk in models adjusted for baseline characteristics and lifestyle factors. Further adjustment for BMI attenuated these associations, although the association with decreasing sleep duration remained statistically significant (HR 1.24, 95% CI 1.03–1.50). These results support considering sleep history alongside diet and physical activity.

## Clinical studies of diabetes pathophysiology and lifestyle in Japan

### Adiponectin and type 1 diabetes

Adiponectin is an adipocyte-derived hormone, and low circulating adiponectin concentrations are associated with visceral fat accumulation and increased metabolic risk. In an analysis of health examination data from 16,892 adults at Sumitomo Hospital, Osaka, Japan, lower serum adiponectin concentrations were associated with a greater number of borderline metabolic abnormalities, even among people without overt diabetes, hypertension, or dyslipidemia [10]. Another analysis of 21,100 Japanese adults without apparent disease found an inverse association between adiponectin concentrations and blood pressure [11]. These observations suggest that low adiponectin concentrations accompany the clustering of metabolic risk factors even in the absence of overt disease.

Alongside these studies, we conducted clinical studies of type 1 diabetes as part of the work of the Japan Diabetes Society Committee on Type 1 Diabetes Mellitus Research. We investigated factors associated with sudden death or cardiac arrest at the onset of fulminant type 1 diabetes, including the severity of hyperglycemia [12]. We also characterized type 1 diabetes associated with anti-programmed cell death-1 therapy in a nationwide survey [13]. In experimental work, we established a model mimicking viral infection using human induced pluripotent stem cell-derived insulin-producing cells, and examined the anti-apoptotic effects of a glucagon-like peptide − 1 analog [14]. Together, these studies provided a foundation for considering diabetes from both clinical and biological perspectives.

### Weight change, fat accumulation, and type 2 diabetes risk

To generate evidence in Japanese populations, we analyzed data from 41,539 adults without diabetes in Kochi Prefecture, Japan, identified using the Kokuho Database, a database of health examination and health insurance claims data [15]. Compared with participants with stable body weight, defined as weight gain or loss of less than 1% over one year, those with weight loss of at least 1% had a lower subsequent risk of type 2 diabetes, whereas those with weight gain of at least 1% had a higher risk. These associations, however, differed by baseline BMI and age. Among participants with a BMI of 22 to less than 25 kg/m^2^, a range below the Japanese threshold for obesity, weight gain of 3% or more was associated with higher risk. In those with a BMI below 22 kg/m^2^, none of the individual weight-change categories was significantly associated with risk, although the trend across categories was statistically significant. No significant association was observed among participants aged 75 years or older. These findings suggest that weight gain merits attention even in people without obesity, and that weight-management goals should be individualized according to age and baseline BMI.

Body weight alone does not capture the distribution of adipose tissue. In a computed tomography study of 84 adults at Osaka University Hospital, we examined the interrelationships among visceral, subcutaneous, and ectopic fat accumulations [16]. After adjustment for age, sex, and BMI, visceral fat showed positive correlations with ectopic fat accumulation, whereas subcutaneous fat showed inverse correlations. Given the metabolic risks associated with visceral and ectopic fat accumulation, these findings highlight the importance of considering fat distribution in addition to BMI.

### Eating behaviors and taste sensitivity as potential intervention targets

Among ectopic fat accumulations, pancreatic fat has gained attention because of its associations with impaired glucose tolerance and type 2 diabetes [17]. In a retrospective study of 185 adults with type 2 diabetes, we investigated pancreatic fat deposition using computed tomography [18]. Among the participants with meal-frequency data, consuming two meals per day was associated with greater pancreatic fat accumulation than consuming three meals per day. In another cross-sectional study of 99 hospitalized adults, fast eating, eating late at night, and early morning awakening were associated with greater pancreatic fat accumulation [19]. These small observational studies suggest that eating and sleep habits are associated with pancreatic fat accumulation. Intervention studies are needed to determine whether changing these habits can reduce pancreatic fat accumulation.

In Japan’s super-aged society, diabetes care for older adults needs to address frailty and health-related quality of life in addition to glycemic control. We assessed taste sensitivity in 70 older adults (mean age, 82 years) living independently in a residential community [20]. Lower sweet taste sensitivity was associated with higher frailty scores and poorer mental health-related quality of life after adjustment for age, sex, and BMI. Although the study was not restricted to people with diabetes, these findings suggest a potential role for assessing taste sensitivity and eating habits when developing tailored lifestyle support for older adults.

## Conclusions and future directions

Our clinical and nutritional epidemiologic studies have identified associations of diet quality and other lifestyle factors with health outcomes relevant to diabetes. Evidence from US cohorts highlights the importance of diet quality and shows that healthful dietary patterns are associated with favorable outcomes. Studies in Japan add information on weight change, fat distribution, eating behaviors, and taste sensitivity. Together, these findings suggest that lifestyle support should be tailored to individual metabolic characteristics, food culture, and living circumstances.

Most of these findings come from observational studies, and residual confounding, measurement error, and limited generalizability should be considered when applying them to clinical practice. It is therefore essential to examine whether changes in diet and other lifestyle behaviors improve health outcomes. Our future work will build on these findings through intervention studies and the implementation of lifestyle support programs in clinical and community settings to advance individualized diabetes prevention and care.
